# Incidence, Risk Factors, Maternal and Neonatal Outcomes of Peripartum Cardiomyopathy (PPCM) in Oman

**DOI:** 10.5334/gh.1198

**Published:** 2023-05-02

**Authors:** Nihal Al Riyami, Safa Al Khayari, Riham Al Zadjali, Lovina Machado, Alya Al Madhani, Hatim Al Lawati

**Affiliations:** 1 Sultan Qaboos University College of Medicine and Health Science, OM; 2 Oman Medical Specialty Board Obstetrics & Gynecology Residency Training, OM; 3 Sultan Qaboos University Hospital, Department of Obstetrics & Gynecology, OM; 4 Royal Hospital, Department of Obstetrics & Gynecology, OM; 5 Sultan Qaboos University Hospital, Department of Medicine, Cardiology, OM

**Keywords:** cardiomyopathies, peripartum period, heart failure, pregnancy, incidence

## Abstract

**Background:**

Peripartum cardiomyopathy (PPCM) is an idiopathic life-threatening condition occurring towards the end of pregnancy or in the first few months following delivery that might affect the maternal and neonatal outcomes.

**Objectives:**

To assess the incidence and to evaluate the antenatal risk factors and the maternal and neonatal outcomes in Omani women diagnosed with PPCM.

**Methods:**

A retrospective cohort study was conducted at two tertiary institutions in Oman between the 1^st^ of January 2010 to the 31^st^ of December 2018. All cases fitting the standard definition of PPCM were included in the analysis. Patients with pre-existing dilated cardiomyopathy, chronic obstructive pulmonary disease and significant valvular heart disease have been excluded.

**Results:**

A total of 113,104 deliveries were screened during the study period. PPCM was confirmed in 116 cases with an incidence of 1.02 per 1000 deliveries. Independent predictors for the development of PPCM were age; especially women at the mid reproductive age (26–35 years), singleton pregnancy and gestational hypertension. In general, maternal outcomes were favorable, with full recovery of left ventricular ejection fraction in 56.0%, recurrence of 9.2%, and an overall mortality rate of 3.4%. The most common maternal complication was pulmonary edema (16.3%). The neonatal mortality rate was 4.3% and the preterm birth rate was 35.7%. Neonatal outcomes included 94.3% live births, out of which 64.3% were term with Apgar scores of more than 7 at five minutes in 91.5% of the neonates.

**Conclusion:**

Our study resulted in an overall incidence of PCCM in Oman of 1.02 in 1000 deliveries. Given the significance of maternal and neonatal complications, establishing a national PPCM database and local practice guidelines, and emphasizing their implementations in all regional hospitals, are fundamental for early recognition of the disease, timely referral, and application of therapy. Future studies, with a clearly defined control group, are highly recommended to appraise the significance of antenatal comorbidities in PPCM compared to non-PPCM cases.

## Introduction

Peripartum cardiomyopathy (PPCM) is an idiopathic life-threatening condition occurring towards the end of pregnancy or in the first few months following delivery [1]. The reported incidence is widely variable, depending on the geographic location. The highest incidence (1 in 102 deliveries) was reported in Nigeria and the lowest (1 in 15,533 births) in Japan. Of note, the incidence is higher in African Americans, African countries, and Haitians, suggesting that the black race is highly susceptible to PPCM [2].

The original PPCM diagnostic criteria, established in 1971, limited the time frame for the diagnosis to between one month antepartum and five months postpartum. However, this was challenged by numerous subsequent publications describing earlier and later development of peripartum cardiomyopathy during pregnancy [3]. The condition is characterized by the development of left ventricular systolic dysfunction with a concomitant reduction in left ventricular ejection fraction (LVEF) to less than 45% [4,5,6]. The reported mortality rates vary from country to country, and in the developing countries, the reported maternal mortality approached 25% within five years of diagnosis [7].

The main associated risk factors include race [6], maternal age beyond 35 years, multiparty and maternal cocaine abuse [3]. Additionally, women with pre-eclampsia are at four times greater risk of developing PPCM than the general population [5].

The exact etiology remains unknown [4]. However, a number of hypotheses have been proposed such as enhanced oxidative stress, and cleavage of prolactin to the cardio-toxic angiostatin N-Terminal 16K DA prolactin fragment, which results in endothelial damage and myocardial dysfunction. Furthermore, the up-regulation of tyrosine kinase leads to impairment of vascular endothelial growth factor (VEGF). Additionally, acute myocarditis, an abnormal maternal immunologic response to fetal antigens and familial predispositions are some other possible causes of PPCM. Certain inflammatory cytokines such as tumor necrosis factor alpha and interleukin 6 levels are increased in PPCM leading to apoptosis signaling receptor, resulting in more severe manifestations [6].

Women with PPCM classically present with clinical signs and symptoms of heart failure, mainly dyspnea, cough, hemoptysis, as well as an elevated jugular venous pressure and mitral regurgitation. Thromboembolism may also be seen in some cases. If PPCM is suspected, an electrocardiogram (ECG) and echocardiogram are usually ordered. ECG abnormalities in PPCM include atrial abnormality, ventricular hypertrophy, as well as ST-segment deviation, and/or bundle branch block. However, normal ECG does not rule out PPCM [8]. A diagnosis of PPCM is confirmed by the exclusion of other underlying disorders and strict echocardiographic indications of LV dysfunction, defined as an LVEF of less than 45% [9]. Elevated brain natriuretic peptide (BNP) in the blood were linked to adverse maternal cardiac events, such as ventricular systolic or diastolic dysfunction [10].

There is very limited data regarding the recommended time and mode of delivery. In general, early delivery is preferred in advanced cases of heart failure due to PPCM [3]. To date, the recommendations on the time and mode of delivery have been limited and inadequate in guiding peripartum management.

A multidisciplinary approach is required to manage PPCM involving high-risk obstetrics, cardiology, neonatology, and intensive care. According to the European Society of Cardiology (ESC), therapies for patients with acute PPCM have been proposed under the BROAD label: bromocriptine, oral heart failure therapies, anticoagulants, vaso-relaxing agents such as nitrate and hydralazine, and diuretics [11]. However, for women who present with severe LV dysfunction >6 months following first presentation despite optimal medical therapy, implantation of an implantable cardioverter defibrillator (ICD), as well as cardiac resynchronization therapy (CRT) are recommended according to current ESC device guidelines. Lastly, cardiac transplantation, albeit an option in advanced disease, was associated with worse outcomes such as inferior graft survival, higher rejection rates, and higher mortality when compared to cardiac transplant recipients for other indications [6].

To the best of our knowledge, our research is the first of its kind to examine the epidemiology and clinical predictors of PPCM in affected women in Oman. The aim of the study was to assess the incidence, clinical risk factors, and maternal and fetal outcomes in women with PPCM managed at two tertiary centers in Oman.

## Methods

A retrospective cohort study was conducted between the 1^st^ of January 2010 to the 31^st^ of December 2018, including all women diagnosed with PPCM who attended Sultan Qaboos University hospital (SQUH) and Royal Hospital (RH) for antenatal care and delivery, or admitted during the postpartum period with PPCM, or seen in the outpatient cardiology clinic as a referral from other regional hospitals with the diagnosis of PPCM. Ethical approval was obtained from both institutions; SQUH (MREC approval # 1857) and Royal hospital (SRC # 57/2019). SQUH and RH are the only tertiary centers in Oman located in Muscat region.

A total of 113,104 deliveries were screened. Data was collected for 160 women with the diagnosis of PPCM in their medical records or registry. The final analysis included 116 cases after excluding all women with pre-existing dilated cardiomyopathy, chronic obstructive pulmonary disease, significant valvular heart disease, myocarditis, or with missing data. Incidence was calculated by computing the total number of new cases diagnosed at the study period per 1000 deliveries in both centers.

Risk factors and outcomes were studied among the total number of cases identified during the study period.

Data was collected from the delivery records and hospital information system (Trak Care) at SQUH and from the maternity registry and electronic medical records (Al-Shifa system) at Royal Hospital. This included maternal demographics such as: age, region of origin, gravidity, parity, and booking details such as blood group, serology for hepatitis B, HIV, and rubella, full blood count, and dating ultrasound. Other parameters were pregnancy details, medical, and antenatal co-morbidities and gestational age at diagnosis.

In addition, clinical characteristics at presentation like vital signs, symptoms and New York Heart Association (NYHA) functional status, and initial investigations such as: serum troponin level, brain naturitic peptide (BNP) level, chest X-ray, electrocardiography (ECG), and echocardiography were collected. A complete list of medications used by the patient after diagnosis was recorded.

The recorded maternal outcomes included mode of delivery, admission to intensive care unit, duration of hospital stay, pulmonary edema, infection, and thromboembolism. In addition, we captured recovery rates, rates of recurrence, and mortality. The recovery was observed when the LVEF was 50% and more.

Regarding the neonatal outcomes, we included gender, birthweight, Apgar scores, and admission to neonatal intensive care unit (NICU).

### Statistical Analysis

– Data was collected on a data collection sheet then entered and analyzed using Statistical Package for Social Science (SPSS) software version 26.
– Incidence was calculated per 1000 deliveries in Oman using Excel program.
– Risk factors and outcome data were analyzed and categorized in frequency table, whereas the continuous data had been shown as mean ± SD or median (IQR).

## Results

**Figure 1:**
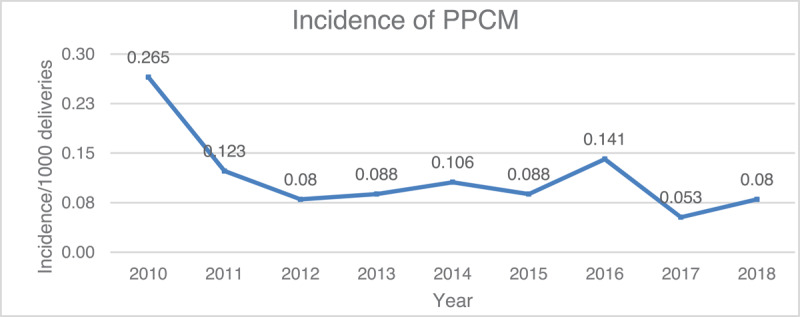
A linear graph demonstrating the incidence of PPCM in Oman in the period from.

Among the 113,104 deliveries, 116 cases were confirmed with isolated PPCM, reflecting an incidence of 1.02 per 1000 deliveries during the eight-year study period. The incidence was higher in 2010 and 2016, at 0.265 and 0.141 per 1000 deliveries, respectively (Figure 1).

### Demographic Features

The mean maternal age at the time of diagnosis was 32.00 ± 5.462 (20–48) years. PPCM was noted more in multiparous women (n = 63, 54.3%) with 78 (67.2%) of the cases had their antenatal care elsewhere, but most of them were referred to specialized clinics after being diagnosed at their regional hospitals.

Geographically, 56 (48.3%) of the cases were from Muscat the Capital Region, 15 (12.9%) from Al Sharqyia, 15 (12.9%) from Al Dakhlyia, and 13 (11.2%) from South Al Batinah. Only one case (0.9%) diagnosed in both Al Dhahira and Dhofar. Two (1.7%) patients had a positive family history with one other female member affected (Table 1).

**Table 1:** Demographic data and pregnancy details.


VARIABLES	OUTCOME OF 116 CASES

Institution, n (%)	

RH	98 (84.5%)

SQUH	18 (15.5%)

Region n (%)	

Muscat	56 (48.3%)

South Al-Batinah	13 (11.2%)

North Al-Batinah	9 (7.8%)

Al-Sharqiyah	15 (12.9%)

Musandam	3 (2.6%)

Buraimi	3 (2.6%)

Dhufar	1 (0.9%)

Al-Dhahira	1 (0.9%)

Al-Dakhiliya	15 (12.9%)

Family history of PPCM	2 (1.7%)

Maternal age (years), n (%)	

20–25 years	14 (12.1%)

26–30 years	32 (27.6%)

31–35 years	37 (31.9%)

36–40 years	28 (24.1%)

>40 years	5 (4.3%)

Parity, n (%)	

One birth	53 (45.7%)

Two or three births	12 (10.3%)

Four or more births	51 (44.0%)

Antenatal booking status, n (%)	

Booked	38 (32.8%)

Un booked	78 (67.2%)

Number of fetuses, n (%)	

Singleton	83 (71.6%)

Multiple pregnancies	10 (8.6%)

Missing	23 (19.8%)

Gestational age at diagnosis, n (%)	

Third trimester	28 (24.1%)

Intrapartum	2 (1.7%)

Postpartum	76 (65.5%)

Missing	10 (8.6%)


SQUH: Sultan Qaboos University Hospital, RH: Royal Hospital, PPCM: Peripartum Cardiomyopathy. * Unbooked: pregnant women who did not follow during pregnancy at SQUH or RH.

### Pregnancy Details

PPCM was more common in singleton pregnancies accounting for 71.6% (n = 83) of the studied population compared to multiple pregnancies, 8.6% (n = 10). Interestingly, PPCM was diagnosed mainly in the postpartum period, 76 (65.5%) of the subjects. It was seen in the third trimester in 28 (24.1%) of the women studied and rarely occurred during the intrapartum period, as it was detected in only two (1.7%) of the patients (Table 1).

### Antenatal Comorbidities

Among PPCM cases diagnosed at the two centers, 12 (10.3%) were found to have gestational diabetes and seven (6.0%) had preexisting diabetes mellitus. In addition, 12 (10.3%) had gestational hypertension, compared to none (7.8%) who had pre-existing chronic hypertension. Moreover, four (3.4%) patients had sickle cell disease, and two (1.7%) had thalassemia major and had a normal cardiac function prior to pregnancy (Table 2).

**Table 2:** Antenatal comorbidities.


ANTENATAL COMORBIDITIES	OUTCOMES N (%)

Diabetes in pregnancy:	

Gestational diabetes	12 (10.3%)

Pre-existing diabetes	7 (6.0%)

Hypertensive disorders of pregnancy:	

Gestational hypertension/pre-eclampsia	25 (21.6%)

Chronic hypertension	9 (7.8%)

Hematological diseases:	

Sickle cell disease	4 (3.4%)

Thalassemia major	2 (1.7%)

Asthma	7 (6.0%)

Hepatitis B	2 (1.7%)

Congenital heart disease	1 (0.9%)


### Clinical Characteristics

The most common reported symptom was shortness of breath at rest in 71 (61.2%) patients. Exertional dyspnea was reported in 45 (38.8%), palpitations in 20 (17.2%), orthopnea in 19 (16.4%), chest pain and cough in 15 (12.9%) patients each, paroxysmal nocturnal dyspnea in nine (7.8%), sweating in four (3.4%), and fever in three (2.6%) patients. Two (1.7%) patients had an arrest during labor one of which was a ventricular fibrillation, while the other had a respiratory arrest and was found to have sinus tachycardia (Table 3).

**Table 3:** Clinical Characteristics.


CLINICAL CHARACTERISTICS PRESENTED AS N (%), UNLESS EXPRESSED OTHERWISE	OUTCOMES (N = 116)

Presenting symptoms, n (%)	

Shortness of breath	71 (61.2%)

Exertional dyspnea	45 (38.8%)

Palpitation	20 (17.2%)

Orthopnea	19 (16.4%)

Chest pain	15 (13.5%)

Cough	15 (12.9%)

Paroxysmal nocturnal dyspnea	9 (7.8%)

Sweating	4 (3.4%)

Fever	3 (2.6%)

Cardiac arrest during labor	3 (2.6%)

Signs, n (%)	

Tachycardia	9 (7.8%)

Bradycardia	2 (1.7%)

Hypotension	1 (0.9%)

NYHA classification	

Class I	34 (29.3%)

Class II	53 (45.7%)

Class III	14 (12.1%)

Class IV	15 (12.9%)

Electrocardiography, n (%) (n=32)	

Sinus tachycardia	10 (33.3%)

Supraventricular tachycardia	0 (0.0%)

ST depression	1 (3.2%)

WPW syndrome	1 (3.3%)

Left ventricular hypertrophy changes	3 (9.4%)

Chest X-ray, n (%)	

Cardiomegaly	16 (17.2%)

Pulmonary congestion	10 (11.0%)

Obliterated costophrenic angles	3 (3.2%)

Pleural effusion	6 (6.7%)

Troponin Value (pg/mL), median (IQR) (n = 22)	11.5 (0.04–37.25)

BNP Value (pg/mL), median (IQR) (n = 9)	2420.00 (373.00–6132)


Based on the New York heart association classification (NYHA classification), 53 (45.7%) patients were categorized as class II, followed by class I in 34 (29.3%) patients, 15 (12.9%) with class IV, and only 14 (12.1%) patients with class III.

Thirty-two patients had ECG done at the time of diagnosis, out of which 15 (46.9%) showed non-specific ECG abnormalities described as follows: sinus tachycardia in 10 (33.3%) patients, left ventricular hypertrophy changes in three (9.4%) patients, ST depression and Wolff-Parkinson-White (WPW) syndrome in one (6.7%) patient each (Table 3).

### Radiological and Laboratory Investigations

Chest X-ray showed cardiomegaly in only 16 (17.2%) out of 116 patients, pulmonary congestion in 10 (11.0%), pleural effusion in six (6.7%), and obliterated costophrenic angle in three (3.2%) patients (Table 3).

Among 116 cases, 22 patients had their serum troponin level measured, with a median of 11.5 pg/mL. On the other hand, only nine patients had their BNP measured at the time of diagnosis, with a median of 2420.00 pg/mL (Table 3).

Echocardiography findings were recorded for 85 patients. Thirty-one patients had their echocardiography done elsewhere and the details were not recorded. Left ventricular ejection fraction (LVEF) for each patient was documented at the time of diagnosis (LVEF1), after six months (LVEF2) and after 18 months (LVEF3). The mean LVEF1 at diagnosis was found to be 33.25% (15-45, SD ± 7.72), 43.3% for LVEF2 at six months (20-65, SD ±12.32), and 45.83% (15-62, SD ± 12.50) for LVEF3 at 18 months (Table 4).

**Table 4:** Echocardiography Changes.


	MEAN% (RANGE, ± SD)	MISSING

LVEF 1	33.25% (15–45, ± 7.72)	8

LVEF 2	43.3% (20–65, ±12.32)	64

LVEF 3	45.83% (15–62, ± 12.50)	13


LVEF 1: Left ventricular ejection fraction at the time of diagnosis. LVEF 2: Left ventricular ejection fraction at 6 months post diagnosis. LVEF 3: Left ventricular ejection fraction at 18 months post diagnosis.

Regarding the medications used at presentation, the data was recorded for 100 patients. Out of which 72 (72.6%) received furosemide, 67 (67.7%) received carvedilol, 47 (47.5%) were on lisinopril, spironolactone prescribed for 35 (35.4%) patients, and only two (2.0%) patients received bromocriptine. One patient (1.0%) was commenced on warfarin following evidence of RV and LV thrombus. Patients were counselled on cessation of breastfeeding prior to medication administration.

### Maternal Outcomes

Out of 73 women with recorded information, 50 (68.5%) patients underwent Cesarean section, whereas 21 (28.8%) delivered spontaneously. Only two (2.7%) patients required instrumental delivery.

Of the cohort, 23 (31.9%) were admitted to the intensive care unit (ICU), out of which 16 patients suffered low oxygen saturation, five patients had pulmonary edema, one patient had a cardiac arrest during labor and one patient was admitted for pulmonary emboli. Details on the length of hospital stay are further demonstrated in Table 5.

**Table 5:** Perinatal Outcomes.


PERINATAL VARIABLES PRESENTED AS N (%), UNLESS EXPRESSED OTHERWISE	PERINATAL OUTCOME (N = 73)

Mode of delivery, n (%) for 73 women	

Spontaneous vaginal delivery	21 (28.8%)

Instrumental vaginal delivery	2 (2.7%)

Cesarean section	50 (68.5%)

Intensive care unit admission, n (%)	23 (31.9%)

Duration of hospital stay, n (%) (n = 35)	

<7 days	19 (54.3%)

7–14 days	13 (37.1%)

15–28 days	1 (2.9%)

29–32 days	2 (5.7%)

Complications, n (%)	

Pulmonary edema	15 (16.3%)

Infection	7 (7.1%)

Thromboembolism	2 (2.0%)

Fully recovered, n (%)	61 (56.0%)

Recurrence, n (%)	8 (9.2%)

Mortality, n (%)	4 (3.4%)

Gestational age at delivery, n (%)	

28 to 33+6 weeks	6 (8.2%)

34 to 37+6 weeks	14 (19.2%)

>37+6 weeks	53 (72.6%)

**NEONATAL OUTCOMES (N = 51)**	

Gender, n (%)	

Male	24 (47.1%)

Female	27 (52.9%)

Apgar score at 5 minutes, n (%)	

0–6	4 (8.5%)

7 or higher	43 (91.5%)

Missing	4

NICU admission n (%)	9 (17.6%)

Live birth, n (%)	49 (94.3%)

Stillbirth, n (%)	2 (4.3%)

Birth weight (mean, range ± SD)	

Male	3.00, (1.80-4.70) ± 0.79

Female	2.76 (1.12-4.4) ± 0.85


The main complications noted were pulmonary edema in 15 (16.3%), infections mainly pneumonia occurred in seven (7.1%), and thromboembolism in two (2.0%) patients. One of which had a right and left ventricular thrombus while the other had a deep vein thrombosis eventually leading to pulmonary emboli. All complications were noted during presentation at the immediate postpartum period.

During follow-up, 61 (56.0%) patients had fully recovered left ventricular function, eight (9.2%) had a recurrence in following pregnancies. The observed maternal death rate was found to be 3.4% (n = 4).

### Neonatal Outcomes

In total, neonatal data collected for 51 cases out of 116 as most of these patients delivered in their regional hospitals. Out of these newborns, 24 (47.1%) were males and 27 (52.9%) were females. Out of the total number of newborns, 50 (94.3%) were born alive and two (4.3%) were stillborn. The mean birthweight for the females was found to be 2.76 (1.12–4.4) ± 0.85 kg, whereas the mean birthweight for the males was reported as 3.00 (1.80–4.70) ± 0.79 kg.

Thirty-six (64.3%) newborns were born at term (<37+6 weeks of gestation), 14 (25.0%) born at the gestational age of 34 to 37+6 weeks and six (10.7%) were born at 28 to 33+6 weeks of gestation.

Forty-three (91.5%) newborns had a good Apgar score of 7 or higher at 5 minutes, whereas four (8.5%) babies were born with Apgar score of 0-6. Moreover, nine (17.6%) got admitted to the neonatal intensive care unit (NICU) (Table 5).

## Discussion

The incidence of PPCM is widely variable worldwide with the highest incidence reported in Nigeria (1 in 100 deliveries) [12] and the lowest incidence of 1 per 15,533 births in Japan [2]. The current study showed that the incidence of PPCM in Oman is estimated to be 1.02:1000 deliveries. It is well established that maternal age of 30 years or more is a well-described independent risk factor for PPCM [13]. In our study, most patients with PPCM were above 30 years of age, and commonly multiparous. In contrast, population-based studies of PPCM from South Korea and the USA, demonstrated that the risk is higher in primiparous women [3,14]. Interestingly, yet another study done in California incriminated multiparty as a risk factor for PPCM [15].

The current study, showed that PPCM in Oman was more common in singleton pregnancies compared to multiple gestation. Similar findings were reported in a population-based study from the USA (2004–2011) [14]. However, conflicting results were shared by other studies from South Korea (2010–2012), California, USA (1995–2004), and Iran identifying multiple gestation as a major predisposing factor [3,15,16,17].

In line with observations from international studies, we demonstrated that PPCM occurred mainly in the postpartum period, around six months following delivery, and to a lesser extent in the third trimester. A significant association was noted between the presence of gestational hypertension and the development of PPCM. These findings were in agreement with previous reports [3,14,15,16,17].

According to a recent publication, the presenting symptoms were highly variable and included fatigue, dyspnea, orthopnea, peripheral edema, palpitations, chest pain, decreased exercise tolerance, and abdominal discomfort due to passive congestion of the liver [12]. In our study, most of the cases presented with shortness of breath and exertional dyspnea. Over half of the studied patients were at NYHA II functional status with only mild exertional dyspnea. Two women sustained an arrest while in labor which led to the diagnosis of peripartum cardiomyopathy.

We observed that chest X-ray findings were quite underwhelming and primarily showed cardiomegaly and pulmonary congestion. This is supported by a recently published case report conducted in Tokyo, Japan, in which chest X-ray mainly revealed pulmonary congestion and increased bilateral pulmonary effusion [9]. Analysis of electrocardiographic findings revealed no specific changes. The most common finding was sinus tachycardia. Voltage criteria for left ventricular hypertrophy, whereas ST depression was observed in only one patient. A recent study demonstrated that 50% of PPCM cases showed a prolonged QTc interval, and correlated tachycardia with poor outcomes [1]. Another study showed abnormal ECG in half including atrial abnormality, ventricular hypertrophy, ST-segment deviation, and/or bundle branch block [8].

Echocardiography data was recovered in most of our studied subjects. Unfortunately, 64 patients did not have their echocardiography conducted at six months post diagnosis, despite regular follow-ups at the clinic. This may be attributable to the long waiting list for echocardiography appointments within the tertiary centers. At the time of diagnosis, the mean LVEF was 33.25% and improved to 43.30% at six months and to 45.83% at 18 months.

The most commonly prescribed medications were furosemide and carvedilol. Bromocriptine was used in two patients only. These findings urge us reinforce on the proper management in all regions in accordance to the ESC guidelines [1]. Special considerations should be taken into account during pregnancy and breastfeeding [18].

In consistency with other studies [3,12]. The majority of women in our study were delivered by Cesarean section, and a few cases were admitted to the intensive care unit. Most of the patients were admitted to the hospital for less than seven days. This was consistent with a recent study conducted in the Middle East in which the median length of admission was seven days [19]. However, the exact length of stay for the majority of the study group was not traceable due to admissions in regional secondary hospitals where the diagnosis of PPCM was made prior to tertiary centers referral.

The most common complication noted in our study was pulmonary edema. However, a recent study showed that thromboembolism was the most common severe complication of PPCM, affecting 6.6% of PPCM in the United States; a similar rate (6.8%) has been reported recently in the EU Observational Research Program worldwide registry [20]. In our study only 3.1% of the women had thromboembolism. This may be explained by the fact that some of our patients received proper anticoagulation.

More than half of the subjects enrolled in the study had a full recovery. This is consistent with the recovery rates in an article aimed to study the six-month outcome of PPCM within the German cohort, which was found to be 52%. Whereas recovery rates in the South African cohort in a six-month time frame was as low as 32% [21]. The recurrence rate in our study group was 9.2%.

Maternal mortality rate in PPCM cases in Oman found to be 3.4%, this is considered low in comparison to a study conducted in South Africa where the mortality rates were as high as 11% over a six-month study period [21]. However, the rates in Oman are higher than those found in a recent study conducted in US, where the overall-in-hospital mortality rate was reported to be 1.3% [3,12]. It is known that mortality rates associated with peripartum cardiomyopathy range from 3% to 40%, depending on geographic location [22].

The neonatal outcomes in terms of intrauterine growth restriction and prematurity were better than what has been reported by other studies [14]. A majority of our newborns were born at term. Following delivery, most neonates had a good Apgar score at five minutes. This result is similar to that found in a study conducted in California where only 3.3% of the neonates had an Apgar score of below 7 at five minutes. The rate of neonates who required admission to the neonatal intensive care unit was 17.6%. A majority of PPCM cases resulted in live babies. In contrast to a study published in India in 2020, where the neonatal mortality rates immediately following birth were as high as 11.11% [23].

### Strengths

To our knowledge, this is the first study in Oman that was conducted to estimate the incidence and examine maternal risk factors and outcomes in PPCM. It is a multicenter study across two tertiary health centers in Oman, both accepting PPCM cases referred from regional hospitals either in antepartum period for delivery by an experienced obstetrician team or for follow-up with specialized cardiology team after delivery.

### Limitations

This was a retrospective study, with all the inherent limitations of this type of study, with a relatively small sample size. In addition, most of the cases were diagnosed in peripheral hospitals then referred to the tertiary centers for continuation of care. Therefore, it is conceivable that the medical data were incomplete for some of the patients.

No control group was included in our study to further examine the significance of the maternal risk factors for this disease compared to the general population. This can be the theme of a larger-scale, prospective nationwide study.

## Conclusion

Our analysis suggests a relatively low incidence of PPCM in Oman. This is based on data from the two main tertiary centers in the country. Our results showed that women at the middle reproductive age (26–35 years), singleton pregnancy, multi-parity, and pregnant women with gestational hypertension were at increased risk of developing PPCM.

In general, there was good maternal outcome, with full recovery noted in 56.0% of the women, recurrence in 9.2%, and death in 3.4%. In addition, neonatal outcomes were favorable when compared to international studies. Future studies including prospective, controlled studies including border nation-wide inclusion are necessary to compare outcomes in this specific group of population compared to the general population.
